# Effect of multiple freeze–thaw cycles on the detection of anti-SARS-CoV-2 IgG antibodies

**DOI:** 10.1099/jmm.0.001402

**Published:** 2021-08-06

**Authors:** Farah M. Shurrab, Duaa W. Al-Sadeq, Fathima Amanullah, Salma N. Younes, Hadeel Al-Jighefee, Nadin Younes, Soha R. Dargham, Hadi M. Yassine, Laith J. Abu Raddad, Gheyath K. Nasrallah

**Affiliations:** ^1^​ Biomedical Research Center, Qatar University, Doha 2713, Qatar; ^2^​ College of Medicine, Member of QU Health, Qatar University, Doha, Qatar; ^3^​ Department of Biomedical Science, College of Health Sciences, Member of QU Health, Qatar University, Doha, Qatar; ^4^​ Infectious Disease Epidemiology Group, Weill Cornell Medicine–Qatar, Cornell University, Qatar Foundation – Education City, Doha, Qatar; ^5^​ World Health Organization Collaborating Centre for Disease Epidemiology Analytics on HIV/AIDS, Sexually Transmitted Infections, and Viral Hepatitis, Weill Cornell Medicine–Qatar, Cornell University, Qatar Foundation – Education City, Doha, Qatar; ^6^​ Infectious Disease Epidemiology Group, Weill Cornell Medicine–Qatar, Cornell University, Qatar Foundation – Education City, Doha, Qatar; ^7^​ World Health Organization Collaborating Centre for Disease Epidemiology Analytics on HIV/AIDS, Sexually Transmitted Infections, and Viral Hepatitis, Weill Cornell Medicine–Qatar, Cornell University, Qatar Foundation – Education City, Doha, Qatar; ^8^​ Department of Healthcare Policy and Research, Weill Cornell Medicine, Cornell University, New York, USA

**Keywords:** antibodies, detection, COVID-19, SARS-CoV-2, IgG, freeze, thaw

## Abstract

Several studies have investigated the effect of repeated freeze–thaw (F/T) cycles on RNA detection for severe acute respiratory syndrome coronavirus-2 (SARS-CoV-2). However, no data are available regarding the effect of repeated F/T cycles on SARS-CoV-2 antibody detection in serum. We investigated the effect of multiple F/T cycles on anti-SARS-CoV-2 IgG detection using an ELISA test targeting the nucleocapsid antibodies. Ten positive and 1 negative SARS-CoV-2 IgG sera from 11 participants, in replicates of 5, were subjected to a total of 16 F/T cycles and stored at 4 °C until tested by ELISA. Statistical analysis was performed to test for F/T cycle effect. None of the 10 positive sera became negative after 16 F/T cycles. There was no significant difference in the OD average reading between the first and last F/T cycles, except for one serum with a minimal decline in the OD. The random effect linear regression of log (OD) on the number of cycles showed no significant trend, with a slope consistent with zero (B=−0.0001; 95 % CI −0.0008; 0.0006; *P*-value=0.781). These results suggest that multiple F/T cycles had no effect on the ability of the ELISA assay to detect SARS-CoV-2 IgG antibodies.

## Introduction

Serum banks are well established as they are considered to be an essential reference for clinical information and research use. However, there are concerns regarding the effect of repeated freeze–thaw (F/T) cycles on the biological entities of serum proteins, including immunoglobulins (Ig) [1–4]. It is suspected that repeated F/T cycles may lead to denaturation or degradation of the antibody of interest [5]. This is critical when it comes to sensitive immunoassays such as ELISA or chemiluminescence automated analysers that detect antibodies in serum or plasma. Therefore, it is recommended to store sera in aliquots to reduce sample exposure to multiple F/T cycles [6]. Although several studies have suggested that repeated F/T cycles have a minimal effect on antibody stability against specific pathogens [2, 7, 8], other researchers are questioning the reliability of the data generated from using such samples [9].

The emergence of the severe acute respiratory syndrome coronavirus-2 (SARS-CoV-2) in late December 2019 in Wuhan, PR China, has led to a global coronavirus disease 2019 (COVID-19) pandemic [10]. Several studies showed the effect of repeated F/T cycles on SARS-CoV-2 RNA stability in throat and nasopharyngeal swabs specimens [11, 12]. However, to the best of our knowledge, the stability of SARS-CoV-2 antibodies after multiple F/T cycles has not been assessed. In this study, we investigated the effect of multiple F/T cycles on SARS-CoV-2 IgG detection in serum by using ELISA targeting the nucleocapsid (N) antibodies.

## Methods

The cohort sera used in this study were part of blood specimens that were collected in a previous nationwide survey to assess the prevalence of SARS-CoV-2 detectable antibodies [13]. This study was approved by the Medical Research Centre (MRC) Ethics Committee (MRC-05-136). The specimens were collected between 26 July and 9 September 2020 and frozen and thawed twice during the previous study before being transferred on ice to our facility at Qatar University, where they were stored once more at −80 °C until they were used in this study in December 2020.

Fifty sera were screened using the EDI novel coronavirus COVID-19 IgG ELISA kit (ref. no. KT-1032, USA) targeting the anti-N SARS-CoV2 IgG [14]. Eleven sera were selected from these, of which 10 were IgG-positive and one was IgG-negative. The latter serum was used as a control. For a more representative comparison between measurements, we selected the positive sera that had broadly different optical density (OD) readings (high, medium and low).

From each of the 11 sera, a total of 40 serum aliquots, 5 µl each, were divided into 8 sets of 5 replicates and subjected to 8 different F/T cycles (3, 4, 6, 8, 10, 12, 14, and 16). The first set was stored at 4 °C during the study as a baseline. The remaining seven sets were subjected to the repeated F/T cycles, with one set of aliquots being stored at 4 °C at a time until all cycles were completed. The serum samples were then tested using the EDI kit and the OD reading at 450 nm was recorded.

The average reading for each serum was estimated and plotted against the number of F/T cycles and versus the cut-off values defining a positive or a negative outcome. The cut-off values were calculated according to the manufacturer’s instructions. An independent *t*-test was conducted to compare the OD measurements of the positive sera and the negative serum. Paired *t*-test were performed to compare the log (OD) of the first cycle to the log (OD) of the last cycle. To adjust for any clustering effect from measurements of the same serum, a random effect linear regression was conducted on the log (OD) versus the number of F/T cycles. A *P*-value of <0.05 was considered statistically significant.

## Results

A total of 438 measurements from 11 sera were available for analysis. Two OD readings were excluded due to manual error during the ELISA run. The mean OD value for the positive serum measurements [*n*=398; mean (sd): 0.69 (0.25)] was significantly higher than that of the negative serum measurement [*n*=40; mean (sd): 0.23 (0.03); *P*-value<0.001].

**Fig. 1.**
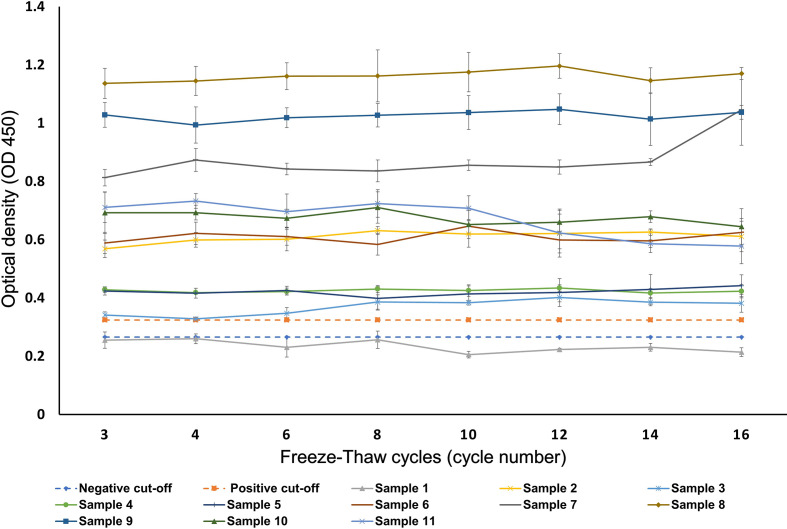
SARS-CoV-2 IgG antibody average 450 Optical density readings of the five replicates for each serum plotted against the number of F/T cycles.

**Fig. 2.**
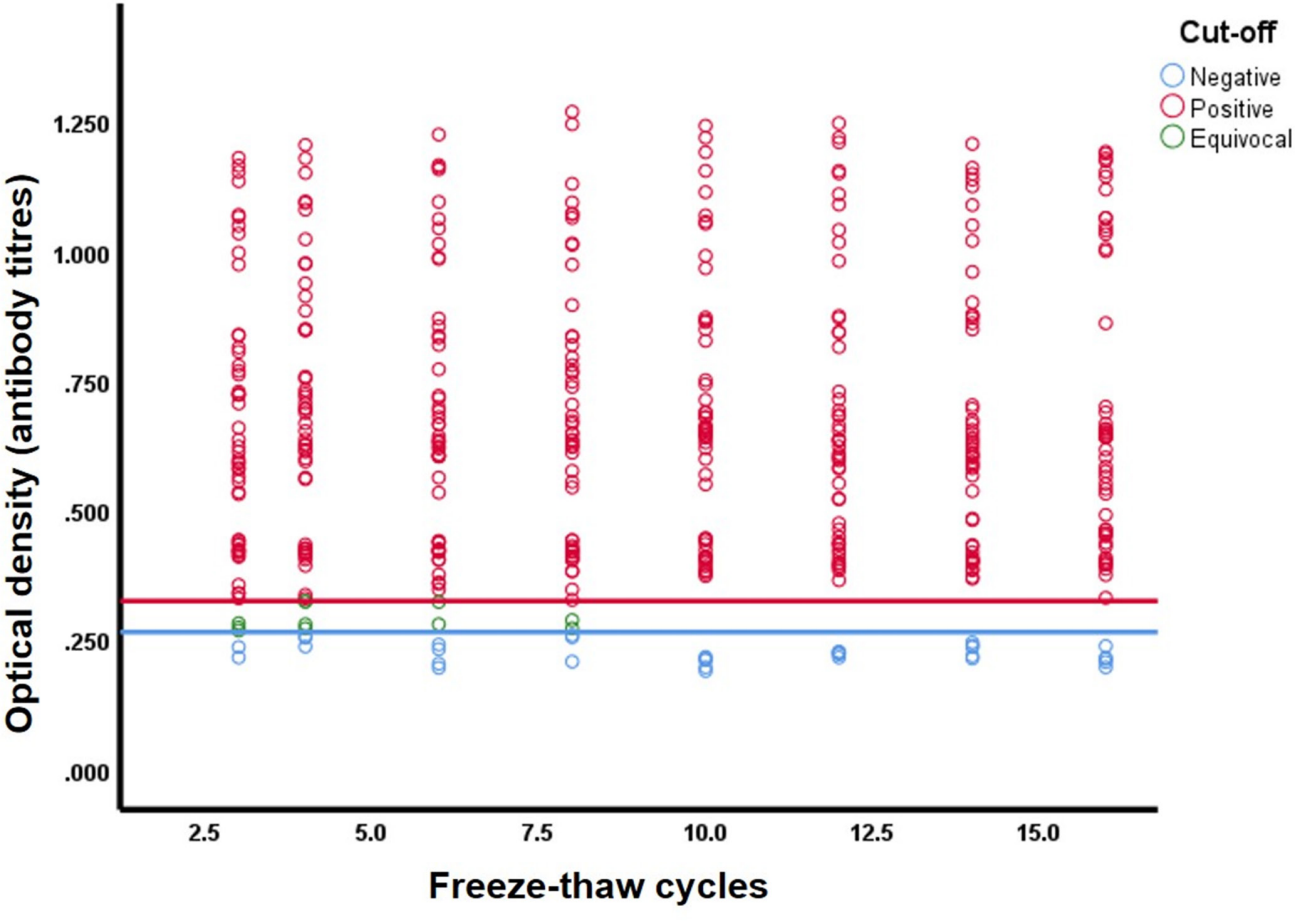
Antibody levels for SARS-CoV-2 for 438 specimens against the number of F/T cycles. The blue and red solid horizontal lines delineate the cut-off for negative at optical density 0.2655 and the cut-off for positive at 0.3245.

The average assay readings for each serum were plotted against the F/T cycles and versus the cut-off values delineated at ≤0.2655 for negatives and ≥0.3245 for positives (Fig. 1). When antibody OD readings were analysed as categorical outcomes (positive, negative and equivocal) across the cycles, out of 398 positive measurements, 0.8 % (3/398; 95 % CI 0.3–2.2 %) were no longer classified as positive using the positive cut-off. Instead, they were equivocal. Out of 40 negative measurements, 20.0 % (8/40; 95 % CI 10.5–34.8 %) were no longer classified as negative using the negative cut-off (Fig. 2). Instead, they were equivocal. None of the sera changed from positive to negative or from negative to positive throughout the cycles.

The random effect linear regression of log (OD) on the number of cycles showed no significant trend, with a slope consistent with zero (B=−0.0001; 95 % CI −0.0008; 0.0006; *P*-value=0.781), indicating that the multiple F/T cycles had no effect on the SARS-CoV-2 IgG antibody titres in the serum.

## Discussion

Frozen serum banks are an important source of scientific and clinical information and are essential for infectious disease and vaccine research. It is commonly believed that antibodies keep their stability if serum is stored below −20C^o^ [9]. Nevertheless, evidence suggests that just one F/T cycle can significantly reduce IgG antibody levels [9]. To date, studies investigating the effects of F/T cycles on the detection of SARS-CoV-2 antibodies have yielded inconsistent results, and the availability of data on the effects of multiple F/T cycles, sample size and storage conditions is limiting.

The present study investigated the effect of 16 repeated F/T cycles on SARS-CoV-2 IgG antibodies in serum. The results showed that in nearly all sera there was no significant difference in sample reactivity between the first and last F/T cycle, while none of the reactive sera became non-reactive after 16 F/T cycles and no false-positive results were obtained (Fig. 2). The random effect linear regression showed no significant trend in sera OD reading, with a slope that is consistent with zero. However, there was one outlier with one serum showing an increased OD reading after cycle 16 (Fig. 1, sample no. 7). This outlier may have been caused by a pipetting error between the ELISA plates.

In agreement with our findings, other studies have tested the effect of repeated F/T cycles on measles, mumps, rubella, syphilis, anti-nuclear antibodies (ANA) and anti-neutrophil cytoplasmic antibodies (ANCA), and found similar results [2, 8, 15]. Although each study applied a different F/T methodology and targeted different antigens, they all concluded that there was no clinically or statistically significant difference in the antibody levels after several F/T cycles. Interestingly, another study has shown that 174 F/T cycles on anti-treponemal sera did not affect the stability, the reactivity of antibodies, or the samples’ quality when tested by a chemiluminescence assay [1]. The data generated from our study and the previous studies provide concrete knowledge regarding antibody stability in serum, which allows the maximum potential use of serum, especially for those that have undergone multiple F/T cycles. Additionally, our generated results can be beneficial for the design of serum banks, where it is important to monitor the integrity of sample components.

We conclude that 16 F/T cycles did not interfere with the detection of SARS-CoV-2 IgG antibodies, and had no effect on assay sensitivity. However, in this study, we tested the effect of multiple F/T cycles on anti-SARS-CoV-2 IgG in serum only. It would be ideal to use plasma samples as well and to test the stability of other anti-SARS-CoV-2 antibody classes such as IgM and IgA. Furthermore, our ELISA test targeted antibodies against SARS-CoV-2 nucleocapsid protein alone, but possibly F/T cycles may interfere with other SARS-CoV-2 proteins, such as the spike protein. Lastly, using an automated serological analyser would be beneficial to reduce pipetting errors.
